# A pseudo-outbreak of *Yersinia enterocolitica* in sputum cultures among 4 hospitalized pneumonia patients in Allegheny County, Pennsylvania, 2024

**DOI:** 10.1017/ash.2026.10381

**Published:** 2026-04-20

**Authors:** Nottasorn Plipat, Jennifer Fiddner, Shannon McGinnis, Mabel Kamweli Aworh, Sameh Boktor, Sameera Sayeed, Lisa Carp, Brian Graper, Louise K François Watkins

**Affiliations:** 1 Bureau of Epidemiology, Pennsylvania Department of Health, Harrisburg, PA, USA; 2 Allegheny County Health Department, Pittsburgh, PA, USA; 3 Bureau of Laboratories, Pennsylvania Department of Health, Exton, PA, USA; 4 Allegheny Health Network, Pittsburgh, PA, USA; 5 Division of Foodborne, Waterborne, and Environmental Diseases, National Center for Emerging and Zoonotic Infectious Diseases, Centers for Disease Control and Prevention, Atlanta, GA, USA

## Introduction

Yersiniosis typically presents as a gastrointestinal illness following consumption of contaminated foods, dairy products, or water.^1^ However, rare extraintestinal manifestations such as pneumonia have been reported.^2,3^ Historically, infections have been understood to occur predominantly in African American infants and Asian children. However, recent data indicate an increasing annual incidence among adults across diverse demographic groups.^4^ On December 18, 2024, a hospital infection preventionist (IP) notified the Allegheny County Health Department (ACHD) of a cluster of *Yersinia enterocolitica* isolates detected in sputum cultures from 4 hospitalized patients admitted for pneumonia.

## Methods

The hospital and public health authorities conducted a joint epidemiological investigation. Patient medical records were reviewed to collect clinical and epidemiological information. Hospital IP and laboratory teams initiated an internal investigation including staff assessments, infection prevention audits, and environmental sampling for culture. The clinical microbiology laboratory reviewed all relevant culture reports from the previous year. Public health officials developed exposure assessment questionnaires for patient interviews and reviewed surveillance databases, including electronic case reporting, and SaTScan^TM^, a spatiotemporal analysis software used for cluster detection.

All isolates were submitted to the Pennsylvania State Public Health Laboratory for whole-genome sequencing (WGS) to assess genetic relatedness, measured by the number of single-nucleotide polymorphism (SNP) differences. The analysis was performed using 2 web-based bioinformatics platforms: (i) the National Center for Biotechnology Information Pathogen Detection^5^ and (ii) FDA GalaxyTrakr (a Galaxy instance supported by GenomeTrakr initiative).^6^

## Results

Four patients were admitted to 4 different hospital wards between November 28 and December 20, 2024. All presented with respiratory symptoms, and none had gastrointestinal symptoms. The patients ranged in age from 72 to 82 years old. All had multiple comorbidities and a history of current or former tobacco use. Three patients had chronic obstructive pulmonary disease (COPD), and 1 had immunocompromising conditions. Two patients had sputum collected within 24 hours after admission, and the other 2 on day 4 of hospitalization; 3 samples were noted to be of poor quality. All cultures showed varying degrees of growth of *Yersinia enterocolitica*. One patient had additional moderate growth of *Streptococcus pneumoniae* in the sputum culture, whereas the other had a rare growth of *Pseudomonas aeruginosa*. All the patients received intravenous antibiotic treatment for pneumonia. Two patients had their treatment regimen adjusted following the report of *Yersinia enterocolitica* in sputum cultures. (In the retrospective review, sputum cultures showing light *Yersinia enterocolitica* and moderate *Streptococcus pneumoniae* prompted narrowing from vancomycin/cefepime to ceftriaxone in one patient. In a separate patient, cultures demonstrated moderate normal respiratory flora with occasional *Y. enterocolitica*, and ampicillin-sulbactam plus doxycycline was changed to trimethoprim-sulfamethoxazole.) After a review of medical records and consultation with a hospital epidemiologist, *Yersinia enterocolitica* was determined to be an unlikely cause of clinical infection and was more consistent with external contamination. The WGS analysis demonstrated that all 4 isolates were genetically indistinguishable (0–2 SNP differences), strongly suggesting a common source.

However, following an extensive hospital investigation, no source was identified. Environmental cultures obtained from the laboratory, patient rooms, medical supply and distribution areas, and unused sputum collection containers were negative for *Yersinia enterocolitica.* The culture plates were from 4 separate lots. None of the staff members provided care to all 4 patients, and none reported any illness. None of the patients had undergone a recent bronchoscopy or had received a blood transfusion. A review of respiratory therapy procedures, sputum collection practices, and laboratory processing protocols revealed no breaches. The laboratory review of sputum cultures performed during the year preceding the first patient’s specimen showed no abnormal trends.

Additionally, the patients had no exposure to high-risk foods, no contact with animals, and no common activity in their communities. All lived within an 11-mile radius of one another, and 3 patients had the same public water source. During the investigation period and the prior month, no *Yersinia enterocolitica* infections or gastrointestinal illness clusters were reported in Allegheny County or nearby counties.

## Discussion

This cluster of *Yersinia enterocolitica* isolated from sputum cultures of 4 hospitalized patients was deemed a pseudo-outbreak, a situation in which positive sputum cultures did not indicate the etiologic agent of the clinical infection.^7^ Furthermore, it is unlikely to represent normal respiratory flora, as most respiratory microbiome studies have not identified *Yersinia* spp. A metagenomic study detected *Yersinia enterocolitica* and reported a higher abundance in patients with COPD than in smokers without COPD.^8^ Nonetheless, these isolates were genetically indistinguishable, suggesting that they reflected common exogenous contaminants rather than similar endogenous origins. Although the common source, either community or healthcare-associated, was not identified, the more likely source was healthcare-associated. The hospital voluntarily submitted FDA MedWatch reports for the collection of containers and culture media.

This report highlights the importance of investigating pseudo-outbreaks with the same rigor as true outbreaks to prevent avoidable treatments, procedures, and healthcare expenditures.^9^ The detection of *Yersinia enterocolitica* in unexpected specimen types from multiple patients within a single facility should prompt a thorough investigation. Early collaboration between healthcare facilities and public health authorities is critical and helpful for distinguishing pseudo-outbreaks from true outbreaks.
